# In-house–validated liquid chromatography–tandem mass spectrometry (LC-MS/MS) method for survey of acrylamide in various processed foods from Korean market

**DOI:** 10.1002/fsn3.56

**Published:** 2013-08-28

**Authors:** Sanghee Lee, Miyoung Yoo, Minseon Koo, Hyun Jung Kim, Meehye Kim, Sung-Kug Park, Dongbin Shin

**Affiliations:** 1 Food Analysis Center, Korea Food Research InstituteSeongnam, Korea; 2 Food Contaminants Division, Korea Food and Drug AdministrationSeoul, Korea

**Keywords:** Acrylamide, food safety, in-house validation, LC-MS/MS

## Abstract

Acrylamide (AA) is a chemical found in starchy foods that have been cooked at high temperatures. The objective of this study is to monitor the levels of AA in a total of 274 samples of potato chips, chips (except potato chips), biscuits, French fries, breakfast cereals, chocolate products, tea, seasoned laver, and nut products sampled in Korean market. These processed foods include (1) potato chips, (2) chips (except potato chips), (3) biscuits, (4) French fries, (5) breakfast cereals, (6) chocolate products, (7) tea, (8) seasoned laver, and (9) nut products. Samples used for this study were cleaned up using HLB Oasis polymeric and Accucat mixed-mode anion and cation exchange solid-phase extraction cartridge. Liquid chromatography–tandem mass spectroscopy (LC-MS/MS) was validated in-house as an efficient analytical method for the routine analysis of AA in various food products. AA was detected with a Fortis dC18 (1.7 μm, 100 mm × 2.1 mm) column using 0.5% methanol/0.1% acetic acid in water as the mobile phase. Good results were obtained with respect to repeatability (RSDs < 5%). The recoveries obtained for a variety of food matrices ranged between 94.5% and 107.6%. Quantification during routine monitoring was sensitive enough to detect AA at a concentration of 10 μg/kg. A total of 274 food samples were analyzed for AA. The AA levels in the food groups were in the following order: potato chips > French fries > biscuits > tea > chips (except potato chips) > seasoned laver > breakfast cereals > chocolate products > nut products. AA was detected at levels ranging from not detectable to 1435 μg/kg.

## Introduction

Acrylamide (AA) is an industrial chemical used in the manufacture of polyacrylamides and has also been detected in a wide range of food products from as low as μg/kg to mg/kg levels. When starchy foods, such as potatoes and cereal products are fried, roasted, or baked at temperatures >120°C, a reaction between asparagine and reducing sugars in the food results in the formation of AA (Mottram et al. 2002; Becalski et al. 2003; Yaylayan and Stadler 2005). In 2002, Swedish researchers first reported that AA was present in some foods, and they raised concerns about the health effects of AA consumption. High doses of AA can cause nerve damage, and prolonged exposure has been linked with the development of cancers, particularly reproductive cancers (Tareke et al. 2000; Song et al. 2008). National and international agencies have carried out risk assessments of AA in food and have concluded that efforts should be made to reduce the AA levels to as low as possible. To adequately assess this risk to humans, AA levels in food products need to be accurately measured and compiled; this will require the development of excellent analytical methods for extraction and quantitation of AA. Due to the particular limitations of gas chromatography–mass spectroscopy (GC-MS) in the quantitation of AA levels, that is, reproducibility and the high detection limit of derivation by bromination, LC-MS/MS is emerging as the method of choice for researchers (Tareke et al. 2002; Govaert et al. 2006).

Some of the data on the AA content in food products have already been published and a database has been established by the Institute for Reference Materials and Measurements (IRMM) of the European Commission and the U.S. Food and Drug Administration (FDA 2006; Joint Research Centre (JRC) European Commission 2008). Databases on the AA contents in various foodstuffs are used to calculate dietary exposure of humans and these can be utilized for risk estimates (Hilbig et al. 2004; Boon et al. 2005; Matthys et al. 2005).

For this study, a survey of AA levels in a wide variety of processed foods obtained from Korean market was performed. The processed foods were as follows: (1) potato chips, (2) chips (except potato chips), (3) biscuits, (4) French fries, (5) breakfast cereals, (6) chocolate products, (7) tea, (8) seasoned laver, and (9) nut products. For this purpose, a previously published LC-MS/MS method was used (FDA 2003).

## Material and Methods

### Chemicals

AA and ^13^C_3_-labeled AA (99% isotopic purity) were obtained from Sigma (St. Louis, MO) and Cambridge Isotope Laboratories, Inc. (Andover, MA), respectively. Maxi-Spin filter tube (0.45 μm, PVDF; Alltech Associates, IL, Deerfield, IL), OASIS HLB cartridge (200 mg/6 mL; Waters, Milford, MA), and Bond Elut Accucat cartridge (200 mg/3 mL; Varian, Palo Alto, CA) were used for sample extraction and purification. Stock solution of AA (1 mg/mL) and ^13^C_3_-AA (0.1 mg/mL) dissolved in distilled water was stored in capped amber vials at 4°C.

### Samples

A total of 274 samples of different brands and batches, representing nine food categories, were purchased from local retail shops and fast food restaurants 1–5 days before the analysis, between March and October 2011. The food samples included 4–12 different brands of each type of food. AA was measured in potato chips, chips (except potato chips), French fries, biscuits, chocolate products, breakfast cereals, tea, seasoned laver, and nuts products.

### Sample extraction

The extraction, purification, and determination of AA were according to the FDA method (FDA 2003; Arisseto et al. 2007). A 1 ± 0.1 g sample was weighed in a 50-mL polypropylene tube, and 1 mL of 200 ng/mL ^13^C_3_-AA solution and 9 mL of water added. After mixing for 20 min on a rotating shaker, the suspension was centrifuged at 5000 rpm for 15 min. The clarified aqueous supernatant (5 mL) was placed in a Maxi-Spin filter tube (0.45 μm, PVDF) and was centrifuged at 6500 rpm for 5 min. The filtrate (1.5 mL) was loaded onto an OASIS HLB solid-phase extraction (SPE) cartridge (200 mg/6 mL), which had been conditioned with 3.5 mL of methanol, followed by 3.5 mL of water. Upon elution with water, the first 2 mL was discarded, and the ensuing portion (1.5 mL) was collected. The obtained portion was passed through a Bond Elut Accucat SPE cartridge (200 mg/3 mL), which had been conditioned with 2.5 mL of methanol, followed by 2.5 mL of water. In this step, the first 0.5 mL of the eluate was discarded, and the ensuing portion (1 mL) was collected.

### LC-MS instrumentation

Analyses were performed on an Agilent 1200 pump LC system (Agilent Technologies, Santa Clara, CA) coupled to a 4000 Q TRAP mass spectrometer instrument equipped with a TurboIonSpray ionization source (AB SCIEX, Foster City, CA). Analytical separation was carried out on dC_18_ Fortis column (100 × 2.1 mm i.d., 1.7 μm; Fortis Tech., Seoul, Korea).

The mobile phase employed for the isocratic elution of the analyte was a mixture of 0.5% methanol/0.1% acetic acid in water, and the flow rate was 100 μL/min. The total run time of the chromatograms was 10 min and the retention time of AA and ^13^C_3_-AA was about 6 min. The injection volume was 10 μL. The column was operated at ambient temperature. Positive ionization was performed using the following settings: ion spray voltage, 5500 V; curtain gas, 25 (arbitrary units); GS1 and GS2, 50 and 60 psi, respectively; and probe temperature, 500°C. The multiple reaction monitoring (MRM) mode was used for ion detection and the transitions from 72 to 55 *m*/*z* of AA and from 75 to 58 *m*/*z* for the deuterated analog were monitored.

### Method validation

The analytical method for AA was validated with regard to selectivity, linearity, limits of detection (LOD), limits of quantification (LOQ), precision, and accuracy.

Matrices that did not contain AA were analyzed to test the selectivity. A blank sample was tested to assess the presence of interference compounds using an identical extraction procedure, including the same chromatographic and spectroscopic settings. These chromatograms were compared with aqueous standard solutions.

Linearity was examined with standard solutions of AA dissolved in distilled water of concentrations of 5, 10, 25, 50, 100, 250, and 500 μg/kg. Linear regression analysis was performed using the ratio of analyte peak area/internal standard peak area versus analyte concentration.

The LOD was based on the standard deviation (SD) of the *y*-intercept of the regression analysis (*α*) and the slope (*S*), using the equation LOD = 3.3 *α*/*S*. The LOQ was calculated using the equation LOQ = 10 *α*/*S*

Precision and accuracy in the matrix were determined by spiking potato crisps with three different concentrations of AA (25, 100, and 500 μg/kg). Three independent analyses were conducted each day in the repeatability tests (*n* = 3, intraday precision) at the three concentrations listed above, and this was repeated for three consecutive days (*n* = 9, interday precision) at the same concentration levels, both of which were assessed as a function of the variation (CV). The accuracy of the method was evaluated based on the recovery of standards from samples.

### Quality control/results verification

The certified reference material (CRM) (potato chips) numbers 108-10-003 (from the Korea Research Institute of Standards and Science, Korea) were also analyzed together with the samples. Some randomly selected samples were spiked with native AA at a concentration of 100 μg/kg.

## Results and Discussion

### Optimization of LC-ESI-MS/MS conditions

Quantitative methods for analyzing AA have been published in peer journals, most of which involve LC-MS/MS analysis (Swiss Federal Office of Public Health 2002; FDA 2003). LC-MS/MS has a high selectivity when working in the MRM mode, in which the transition from a precursor ion to a product ion is monitored. To obtain the selectivity needed to help lower or overcome the background signals, the MS experiment was performed in the MRM mode.

**Figure 1:**
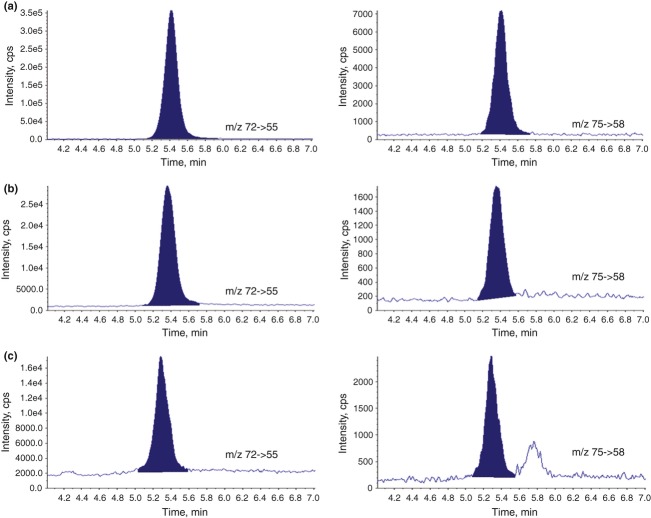
Liquid chromatography–tandem mass spectrometry (LC-MS/MS) chromatograms of a standard solution (100 μg/kg) (a), typical potato chips sample (b), and green tea sample (c). Test sample (left), ^13^C_3_-acrylamide internal standard (right). In the case of test samples, the sample transition of 72–55 *m*/*z* for ^13^C_3_-acrylamide and a transition of 75–58 *m*/*z* for acrylamide were monitored.

The MRM transitions *m/z* 72→55 were acquired for detecting AA (Fig. 1). An additional trace that can be used to monitor AA is *m/z* 72→44 (data not shown). The internal standard, ^13^C_3_-AA was recorded using *m/z* 75→58. AA in food was confirmed if at least two positive MRM responses were obtained at matching ion ratios within an acceptable tolerance (mean ± 10–20%) versus the ratios obtained from standard solutions of AA (Commission Decision 2002/657/EC 2002).

### Sample cleanup

Recently published methods to improve AA recovery throughout sample pretreatment and to concomitantly reduce the amount of coextractives in difficult matrices were utilized as either a single-cartridge or tandem-cartridge combination for cleanup, including Oasis® HLB with nonpolar C8 and C18 phases, Oasis® MAX with an anion exchanger phase, Oasis® MCX with a cation exchanger phase, Isolute Multimode® (Waters Corp., Hilford, MA) with a nonpolar C18 and ion exchanger mixed phase, Bond Elut-Accucat® (Agilent Technology Inc., Lake forest, CA) with a mixed phase, and Oasis® HLB in combination with Oasis® MAX or Oasis® MCX or Bond Elut-Accucat® before LC-MS/MS detection (Rosen and Hellenas 2002; Roach et al. 2003; Leung et al. 2003). In this method, a hydrophilic/lipophilic sorbent (Oasis HLB, 6 cm^3^/200 mg) is applied first to remove most of the sample components. The sample is then passed through a second tube that contains a mixed-mode sorbent (Varian Bond Elut-C18, 3 cm^3^/200 mg) that refines the AA extract. These methods have been shown to provide the best cleanup for analysis (Govaert et al. 2006).

### Method validation

#### Linearity, LOD, and LOQ

Linearity was estimated by investigating the detection signals as a function of analyte concentration with the aid of a regression line using the least squares method. The AA response was linear over a concentration range 5–500 μg/kg with correlation coefficients (*r*^2^) >0.9993. The LODs and LOQs of the proposed methods were calculated on the basis of 3.3 and 10 *σ*/*S*, respectively (*σ*, the standard deviation of the *y*-intercepts of the regression analysis and *S*, the slope of the calibration curve). The LOD and LOQ were 3.2 and 9.6 μg/kg, respectively.

#### Precision and accuracy

The precision based on intraday repeatability was evaluated using replicate (*n* = 3) measurements from standard working solutions at three different concentrations. The interday precision was determined using samples at the same concentration range as mentioned above. A triplicate determination of each concentration over a period of three consecutive days was performed using the same experimental procedures. The method exhibited excellent precision, as shown in Table 1. The intraday data (repeatability) resulted in low relative standard deviation (RSDs) < 3.9%. The interday data conducted on three different days also displayed good values (RSDs < 5.0%), confirming the excellent within-laboratory reproducibility of the method. Both the repeatability and within-laboratory reproducibility obtained using this approach met the criteria of the European Commission Decision (Commission Decision 2002/657/EC 2002).

**Table 1:** Intraday and interday precision data of acrylamide in potato chips

		RSD (%)	
		
Spiking level (μg/kg)	Recovery (%)	Intraday (*n* = 3)	Interday (*n* = 9)
25	92.6	3.9	3.5
100	92.8	1.3	5.0
500	96.4	1.1	3.3

RSD, relative standard deviation.

Accuracy was tested by analyzing CRM (108-10-003 potato chips) supplied by KRISS (Kim et al. 2010). The assigned value of the material was 45.5 μg/kg AA with a satisfactory range 44.3–46.7 μg/kg. The average and their SD were 45.0 ± 0.3 μg/kg. This value was within the satisfactory range for the test material.

#### Spiked recovery

Various types of foods spiked at 100 μg/kg of AA standard solution were analyzed to determine recoveries in different matrices. Recovery data for the analysis of the spiked samples are summarized in Table 2. Excellent recoveries (94.5–107.6%) were obtained with acceptable variation (RSD%, 0.2–3.7%), which fulfilled the legislation requirements. According to EU legislation, the recovery of a confirmatory method should be between 80% and 110% for samples (Commission Decision 2002/657/EC 2002; Van Vyncht et al. 2002).

**Table 2:** Mean recoveries for a range of matrices as determined by standard addition of ^13^C_3_-labeled acrylamide at a level of 100 μg/kg

Matrix	Original (μg/kg)	Spiked (μg/kg)	Determined (μg/kg)	Recovery (%)^1^
Potato chips	53.2	100	151.2	98.0 ± 0.8
Chips (except potato chips)	<LOQ	100	97.7	97.7 ± 1.0
Biscuits	21.7	100	119.3	97.6 ± 2.7
French fries	35.7	100	135.3	99.5 ± 3.2
Chocolate products	<LOQ	100	96.7	96.7 ± 3.2
Breakfast cereals	9.7	100	117.3	107.6 ± 0.7
Tea products	13.3	100	107.8	94.5 ± 0.2
Seasoned laver	<LOQ	100	102.7	102.7 ± 3.7
Nuts products	<LOQ	100	105.0	105.0 ± 1.6

1 Values are mean ± standard deviation of triplicates.

#### Determination of AA in processed foods

The validated method was used to analyze AA contents in 274 food samples from nine food groups, including (1) potato chips, (2) chips (except potato chips), (3) biscuits, (4) French fries, (5) breakfast cereals, (6) chocolate products, (7) tea, (8) seasoned laver, and (9) nut products. Food groups were chosen for analysis if they were previously reported to contain AA or they represented a significant part of the diet. As shown in Table 3, the levels of AA varied considerably between samples in each food group, which was demonstrated by the range in the minimum and maximum values. Mean AA levels in the food groups were in the order of potato chips (596 μg/kg) > French fries (372 μg/kg) > biscuits (250 μg/kg) > tea (245 μg/kg) > chips (except potato chips) (176 μg/kg) > seasoned laver (114 μg/kg) > breakfast cereals (82 μg/kg) > chocolate products (61 μg/kg) > nut products (29 μg/kg).

**Table 3:** Acrylamide contents (μg/kg) in 274 processed food samples covering nine product types from the Korean market analyzed by the LC-MS/MS

		Acrylamide (μg/kg)^1^			
		
Food group	*n*	Mean	SD	Minimum	Maximum
Potato chips	29	596	388	14	1435
Chips (except potato chips)	30	176	182	<LOQ	450
Biscuits	50	250	221	<LOQ	861
French fries	40	372	220	93	918
Chocolate products	20	61	71	<LOQ	232
Breakfast cereals	40	82	82	12	370
Tea products	25	245	314	<LOQ	889
Seasoned laver	20	114	110	<LOQ	335
Nuts products	20	29	40	<LOQ	135

1 Values are mean ± standard deviation of triplicates.

Monitoring studies have been performed on the level of AA in various foodstuffs worldwide. The maximum amount of potato crisps was 1500 μg/kg in Austria (Murkovic 2004), 2528 μg/kg in the Brazilian market (Arisseto et al. 2007), and 3647 μg/kg in Poland (Mojska et al. 2006). In Korea, most potato chips analyzed during 2006–2007 showed the level of AA to be between 195 and 4002 μg/kg (Kim et al. 2009). In our present study, the maximum amount of AA was 1435 μg/kg that is less than Brazilian, Polish, and previously reported data from Korea, but similar to Austrian data. Some studies showed that various cooking conditions such as type of frying oil, frying period, and temperature as well as raw material itself, the potato, and location, climate, and storage of the potatoes affect the level of AA in foodstuffs (Taeymans et al. 2004; Elmore et al. 2005; Palazoglu 2008; Ozkaynak and Ova 2009).

The AA concentration in nonpotato-based chips was lower compared with that of potato chips. Thus, AA accumulation appears to be relatively high in potatoes due to high-temperature cooking relative to other carbohydrate-based materials (Taeymans et al. 2004).

The AA concentrations in nut products and seasoned lavers were not high. The tea of various roasted grains, such as *Polygonatum odoratum*, *Cassia tora*, buckwheat, and barley grain, is mostly consumed in Korea. The amounts of AA quantified in some roasted grain tea were high (889 μg/kg) when compared with levels reported by Ono et al. (2003). According to a previous study, the roasted barley grains for “Mugi-cha” tea, usually processed at 350–400°C for a few minutes, contain 200–600 μg/kg AA (Ono et al. 2003). Therefore, the roasting time and temperature affect the AA level in tea.

The association of AA levels in potato chips and tea suggests that it may be possible to reduce AA levels in some foods through processing changes.

## Conclusion

We determined the quantitative analysis of AA in processed Korean foods using LC-MS/MS. A total of 274 samples, including more than nine different types of foods prepared in various ways, were analyzed. Especially, the monitoring of AA level in tea and seasoned laver produced in Korea has been reported for the first time in this paper. AA concentrations in the different food products analyzed ranged between not-detectable concentrations and 1435 μg/kg.
